# Single-cell transcriptomic analysis uncovers diverse and dynamic senescent cell populations

**DOI:** 10.18632/aging.204666

**Published:** 2023-04-19

**Authors:** Noah Wechter, Martina Rossi, Carlos Anerillas, Dimitrios Tsitsipatis, Yulan Piao, Jinshui Fan, Jennifer L. Martindale, Supriyo De, Krystyna Mazan-Mamczarz, Myriam Gorospe

**Affiliations:** 1 Laboratory of Genetics and Genomics, National Institute on Aging (NIA) Intramural Research Program (IRP), National Institutes of Health (NIH), Baltimore, MD 21224, USA

**Keywords:** senescence, single-cell analysis, transcriptome

## Abstract

Senescence is a state of enduring growth arrest triggered by sublethal cell damage. Given that senescent cells actively secrete proinflammatory and matrix-remodeling proteins, their accumulation in tissues of older persons has been linked to many diseases of aging. Despite intense interest in identifying robust markers of senescence, the highly heterogeneous and dynamic nature of the senescent phenotype has made this task difficult. Here, we set out to comprehensively analyze the senescent transcriptome of human diploid fibroblasts at the individual-cell scale by performing single-cell RNA-sequencing analysis through two approaches. First, we characterized the different cell states in cultures undergoing senescence triggered by different stresses, and found distinct cell subpopulations that expressed mRNAs encoding proteins with roles in growth arrest, survival, and the secretory phenotype. Second, we characterized the dynamic changes in the transcriptomes of cells as they developed etoposide-induced senescence; by tracking cell transitions across this process, we found two different senescence programs that developed divergently, one in which cells expressed traditional senescence markers such as *p16* (*CDKN2A*) mRNA, and another in which cells expressed long noncoding RNAs and splicing was dysregulated. Finally, we obtained evidence that the proliferation status at the time of senescence initiation affected the path of senescence, as determined based on the expressed RNAs. We propose that a deeper understanding of the transcriptomes during the progression of different senescent cell phenotypes will help develop more effective interventions directed at this detrimental cell population.

## INTRODUCTION

Cellular senescence is a state of persistent cell cycle arrest of metabolically active cells that occurs in response to sublethal damage triggered by stimuli from outside and inside the cell [1, 2]. Senescent cells are characterized by altered gene expression programs that orchestrate key senescence-associated traits, such as resistance to apoptosis, an enlarged and flattened cell morphology, dysfunctional cellular organelles, and enhanced lysosomal function with increased senescence-associated (SA) β-galactosidase activity. In addition, they display a senescence-associated secretory phenotype (SASP) through which they secrete proinflammatory cytokines, growth factors, and tissue-remodeling enzymes [3–5]. Despite these shared traits, the manifestation of specific senescence features differs depending on the type and intensity of the senescence-triggering stimulus, the time elapsed since induction of senescence, the type and metabolic status of the cell, and the biological context. Accordingly, senescence is believed to be a highly heterogenous and dynamic developmental process [6].

With recognition that senescent cells accumulate in tissues and organs with advancing age, there is an increased appreciation that senescent cells are largely detrimental during aging, exacerbating age-associated disability and disease [6, 7]. Accordingly, selective removal of senescent cells with senolytic drugs has been shown to extend health span and improve age-associated disorders in mouse models, pointing to the value of senescent cells as therapeutic targets [8–11]. However, the task of identifying senescent cells has been challenging due to the heterogeneity of the senescent phenotype, which makes it difficult to find robust and consistent markers [5, 12, 13]. Thus, a comprehensive analysis of the senescent transcriptome at the individual-cell level will be immensely valuable.

Here, we used single-cell RNA sequencing (scRNA-seq) analysis to document both the diverse transcriptomes of human senescent fibroblasts at an individual-cell scale, and the changes in the transcriptome over time during etoposide-triggered senescence. We have applied this approach using different senescence-triggering stimuli: replicative senescence (RS, recapitulating the process exhaustion of cell division), and DNA damage inducers such as ionizing radiation (IR, used in cancer radiotherapy) and etoposide (ETO, an anti-cancer chemotherapy drug). Analysis of these cells revealed that RS populations displayed broad heterogeneity of transcriptomic patterns, while cells undergoing IR- and ETO-induced senescence showed gene expression patterns that were more similar to each other. By tracking the transitions during senescence initiation and progression in these paradigms, we uncovered the development of divergent senescence programs. Furthermore, we characterized two senescence paths with strikingly different transcriptomic profiles: one exhibiting increased levels of *CDKN2A* mRNA (encoding the tumor suppressor and senescence driver p16), and another characterized by dysregulation of splicing and expression of long noncoding RNAs (lncRNAs). These results advance our understanding of the complexity of senescence phenotypes and illustrate the different transcriptomic paths that characterize senescence programs.

## MATERIALS AND METHODS

### Cell culture and treatments, SA-β-Gal activity, and BrdU incorporation

Human diploid WI-38 fibroblasts (NIGMS Human Genetic Cell Repository at the Coriell Institute for Medical Research; Repository ID AG06814-N) were cultured in Dulbecco’s modified Eagle’s medium (DMEM, Gibco) supplemented with 10% FBS, 1% antibiotics, and 1% non-essential amino acids (Gibco). Cell cultures were maintained in an incubator at 37° C and 5% CO_2_. For replicative senescence (RS), cells were cultured until replicative exhaustion [reaching population doubling level (PDL) > 50]. To induce senescence by treatment with ionizing radiation (IR), proliferating (PDL 24) fibroblasts were exposed to 10 Gray (Gy) and incubated for an additional 10 days. For etoposide (ETO)-induced senescence, cells were treated with either DMSO or 50 μM ETO for six days, then cultured in regular medium without DMSO or ETO-containing medium for four additional days. In time course experiments, cells were collected at 0 (untreated), 1, 2, 4, 7, and 10 days after ETO treatment. For synchronization in G0/G1, cells were washed twice in 1× PBS and cultured in media containing 0.2% FBS for 48 h; cells were then treated with DMSO or ETO with DMSO or ETO.

Senescence was confirmed by staining for senescence-associated β-galactosidase (SA-β-Gal) activity and by measuring BrdU incorporation. SA-β-Gal activity was measured following the manufacturer’s instructions (Cell Signaling Technology). Briefly, cells were washed with 1× PBS, fixed for 10 min at room temperature, and stained overnight with a freshly prepared X-Gal solution (pH 6.0). Micrographs were acquired by using a camera (Nikon Digital Sight) adapted to a microscope (Nikon Eclipse TS100). BrdU incorporation was measured by using the BrdU Cell Proliferation Assay Kit (Cell Signaling Technology). Briefly, cells were incubated in media containing BrdU for 24 h, then fixed and denatured before the addition of the detection antibody. An anti-mouse IgG HRP-linked antibody was used to recognize the bound antibody; the HRP substrate TMB was added to develop color. We halted the development of color by adding the STOP solution and measured BrdU incorporation using a GloMax plate reader (Promega).

### 10x Genomics single-cell library construction, and RNA-sequencing

Single-cell libraries were prepared in two repeats per senescence model and one per time point of the ETO time course experiments using Chromium Next GEM Single Cell 3ʹ Kits v3.1 with Chip G (10x Genomics). In short, collected cells were counted and suspensions of ~7,000 single cells were loaded onto the Chromium Controller Instrument for generation of gel bead-in-emulsions (GEMs). The captured cells were lysed, and the RNA was barcoded while reverse-transcribed in each GEM. GEMs were then broken and the synthetized cDNA was used for library preparation following the manufacturer’s protocol. cDNA quality was assessed on the Agilent Bioanalyzer with High-Sensitivity DNA kit (Agilent). The libraries were sequenced on an Illumina NovaSeq 6000 sequencer with 45,000 to 100,000 mean reads per cell. RNA-seq data were deposited in GEO (GSE226225).

### Quality control and sample processing

10x sequencing libraries were converted into feature-barcode matrices following the Cell Ranger (10x Genomics, version 5.0.0) pipeline. After demultiplexing, the function ‘cellranger count’ was used with default parameters, with the argument ‘include-introns’. Reads were mapped using the Cell Ranger prebuilt annotation platform GRCh38. Subsequent analysis of obtained read count matrices was carried out using the R package Seurat, version 4.1.0 [14]. Applying quality control measures, cells were included for analysis based on the proportion of mitochondrial RNA (<12%), quantity of RNA reads (between 1,000 and 120,000 transcripts for senescent versus control and between 1,200 and 120,000 transcripts for ETO time course analysis), and number of expressed genes (at least 300 independent genes per cell). For the senescence model, one control sample was found to be of a poor quality and was excluded from the analysis.

Each sample was normalized with the ‘LogNormalize’ method using the ‘NormalizeData’ function, and the top 2000 highly variable genes were selected from individual samples with the function ‘FindVariableFeatures’. For reliable single-cell analysis and comparison across conditions, we integrated the individual 10x libraries using the R package Seurat via the pipeline recommended by the developers. Integration anchors were selected with the function ‘FindIntegrationAnchors’, and the samples were integrated using the function ‘IntegrateData’. The integrated dataset was used for dimensional reduction, clustering, and downstream analysis. The ‘RNA assay’ was used when comparing gene expression. Cell cycle scores were assigned using the function ‘CellCycleScoring’ with the cc.genes.updated.2019 dataset provided by Seurat.

### Dimensional reduction and clustering

Dimensional reduction and clustering analysis of integrated data followed the standard Seurat workflow. First, the function ‘ScaleData’ was used to scale the expression values of each gene in integrated data and then the principal component analysis (PCA) implemented with the ‘RunPCA’ function was performed. The top 20 principal components, determined by the ‘ElbowPlot’ method, were applied for two-dimensional data visualization with the uniform manifold approximation and projection (UMAP) via ‘RunUMAP’ function; 20 dimensions were used to find nearest neighbors with default parameters. Clusters were identified using the Louvain algorithm with a resolution of 0.25 when comparing senescence models and a resolution of 0.35 when analyzing the ETO time course. Cluster identities were kept but reordered to aid comparison.

### Genes encoding differentially abundant RNAs

Differentially expressed genes for each senescence model were identified by comparing normalized expressed RNAs for each model to RNAs expressed in control cells via the function’ FindMarkers’ and then merging the results. When comparing clusters, the function ‘FindAllMarkers’ was employed to find marker genes that characterized a specific cluster compared to all other clusters. Genes (RNAs) with absolute average log fold change > 0.25, expressed in minimum 0.25 fraction of a cluster, and with adjusted p-value < 0.05, were retained as marker genes. Overlapping gene numbers among clusters were visualized using UPsetR software.

### Gene set enrichment analysis and process scoring

Gene set enrichment analysis (GSEA) of Gene Ontology (GO) terms employed the ClusterProfiler (version 4.0.5) [15] function ‘gseGO’ on cluster marker genes and their average log fold-change values to find enriched biological processes. Significant processes were defined as having adjusted p-value < 0.05. To reduce the redundancy of terms, the function ‘simplify’ was used with a similarity cutoff of 0.7 when comparing senescence models, and 0.6 when analyzing the ETO time course. The GSEA of “REACTOME_MRNA_SPLICING” gene set from Molecular Signature Database [16] was evaluated using the ‘GSEA’ function. In process scoring, each cell was scored for expression of a gene set representing GO terms selected from GSEA. Genes associated with each term were taken from all clusters for which the term was significantly enriched. The Seurat function ‘FindMarkers’ was applied comparing combined senescent datasets to control cells using these genes. Enrichment scores for senescent cells were selected and used for scoring via the Seurat function ‘AddModuleScore’.

### RNA velocity and splice ratio

For measurement of RNA velocity, the bam files generated from each sample by Cell Ranger were used to create loom files with counts divided into spliced, unspliced, and ambiguous RNA by the standard Velocyto.py (version 0.17) pipeline [17]. In short, the function ‘Velocyto run’ was applied in command line using filtered-feature-barcode matrices, position-sorted (possorted) bam files, and the GRCh38 annotation platform. Loom files were converted to Seurat objects using the ‘as.Seurat’ function in the SeuratObject package. Cell barcodes in these matrices needed to be converted to match their Cell Ranger-generated counterparts. Data from the spliced, unspliced, and ambiguous RNAs were transferred from Velocyto-generated objects to the original objects for further analysis. Splice ratios were calculated from the number of unspliced transcripts relative to total RNA content (spliced + unspliced + ambiguous). RNA velocity was estimated using the function ‘RunVelocity’ included in the SeuratWrappers (version 0.3.0) package using parameters described in the Seurat developers’ vignette (kcells = 25, fit.quantile = 0.02, deltaT = 1, reduction = “umap”). To preserve dynamic changes in population trends, this function was run on two-day intervals instead of the entire integrated dataset. Velocity estimates were visualized using the Velocyto.R (version 0.6) function ‘show.velocity.on.embedding.cor’.

### RT-qPCR analysis

RNA was isolated using phenol-chloroform according to the manufacturer’s instructions (TriPure™ Isolation Reagent, Sigma-Aldrich). Total RNA (500 ng) was used for reverse transcription (RT) followed by quantitative (q)PCR analysis. RT was performed using the Maxima Reverse Transcriptase protocol (Thermo Fisher) and qPCR analysis was carried out using specific primer pairs and SYBR green master mix (Kapa Biosystems) with a QuantStudio 5 Real-Time PCR System (Thermo Fisher). Relative RNA levels were calculated by normalizing to *GAPDH* mRNA, encoding the housekeeping protein GAPDH, using the 2^-ΔΔCt^ method.

## RESULTS

### Heterogeneity of expressed transcriptomes in different WI-38 fibroblast senescence models

**Figure 1:**
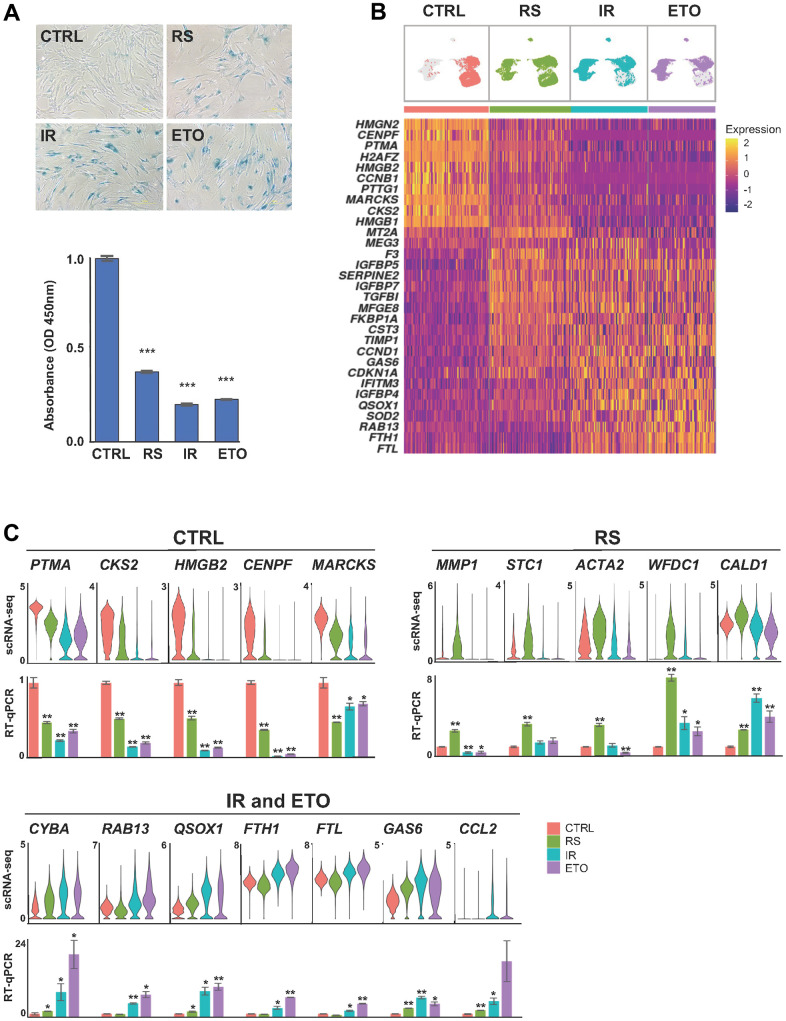
**Shared and distinct transcriptomes of WI-38 fibroblasts in different senescence models.** (**A**) WI-38 fibroblasts that were proliferating at PDL 24 (CTRL), cultured until replicative exhaustion at PDL 57 (RS), exposed to 10 Gy of ionizing radiation and cultured for an additional 10 days (IR), or cultured for six days in media containing 50 μM of etoposide and in regular media for another four days (ETO), were analyzed for SA-β-Gal activity (top), and proliferation rates evaluated by measuring by BrdU incorporation (bottom) and compared to the CTRL population. (**B**) Top, UMAP plots with distribution of cells that were color-coded for each group. Bottom, heatmap showing the relative expression of top marker mRNAs in each population. (**C**) The expression levels of representative top markers from scRNA-seq data (top) were validated by RT-qPCR analysis (bottom). The relative levels of each mRNA in CTRL, RS, IR, and ETO were first normalized to *GAPDH* mRNA levels and each senescence model was compared to CTRL cells. In (**A**, **C**) significance was assessed by two-tailed unpaired Student’s *t*-test, n = 2, *p < 0.05, **p < 0.01, ***p < 0.001.

To better understand the different molecular processes that underlie the heterogeneity of cellular senescence, we comprehensively investigated the single-cell transcriptomes of human diploid fibroblasts (WI-38) exposed to different senescence-triggering stimuli. Proliferating cells (CTRL) at population doubling level 24 (PDL 24) were grown until their replicative potential was exhausted (PDL 57) and they reached replicative senescence (RS). To establish senescence by exposure to ionizing radiation (IR), proliferating cells were exposed to 10 Gy IR, then cultured for 10 additional days. To establish senescence by exposure to etoposide (ETO), cells were cultured in media containing 50 μM ETO for six days and then cultured in regular media for another four days, as described [18]. Cell senescence and proliferative arrest were confirmed by the presence of increased SA-β-Gal activity and decreased BrdU incorporation, respectively (Figure 1A). To measure gene expression profiles at the single-cell level, each sample was processed using single-cell RNA-sequencing (scRNA-seq) analysis following the 10x Genomics pipeline. Out of a total of 27,827 cells, quality control filtering (Material and Methods) retained 20,299 cells for downstream analysis. We framed transcriptomic heterogeneity across all senescence models by integrating the individual cell RNA expression profiles from each condition (CTRL, RS, IR, and ETO) into one dataset.

First, we sought insight into the differences in gene expression patterns across cells from each senescence model. UMAP analysis of the integrated data revealed broad transcriptomic heterogeneity across RS cells that vastly overlapped with CTRL, IR, and ETO samples (Figure 1B). In contrast, IR and ETO cell transcriptomes were more similar to one another and were different from the CTRL group. Interestingly, transcripts encoding cell cycle and proliferation proteins (e.g., *CCNB1, PTMA*, *CKS2,* or *HMGB1/2* mRNAs) were lower in some of the RS cells and in most of the IR and ETO cells, in keeping with the broader transcriptomic diversity of the RS cell populations (Figure 1B and Supplementary Table 1). Markers commonly associated with cellular senescence, including *CDKN1A*, *SERPINE2*, *IGFPB5/7*, *TGFBI*, and *CCND1* mRNAs were upregulated in most cells from all senescence models when compared to proliferating (CTRL) cells. Other transcripts that were highly expressed in all senescence models, included *RAB13*, *TIMP1*, *IGFBP5,* and *QSOX1* mRNAs. Accordingly, GSEA of GO gene sets showed enrichment of cell cycle and proliferation markers in CTRL cells, increased markers of extracellular matrix organization and cell adhesion in RS, as well as upregulation of autophagy, mitochondria and oxidative stress markers in IR and ETO cells (Supplementary Figure 1A). The expression levels of mRNAs from select top marker genes representing each experimental condition relative to CTRL cells by scRNA-seq analysis were validated by RT-qPCR analysis employing primer pairs designed specifically to amplify each mRNA. For validation by RT-qPCR analysis, the mRNAs chosen from the CTRL group were those most highly reduced in the three senescence models; for the RS validation group, the mRNAs chosen were those selectively elevated in this group relative to the CTRL, IR, and ETO groups; and given the extensive overlap between IR and ETO models, the abundant mRNAs preferentially expressed in both models were chosen for validation (Figure 1C and Supplementary Table 2).

### Transcriptomic patterns across different senescence models

**Figure 2:**
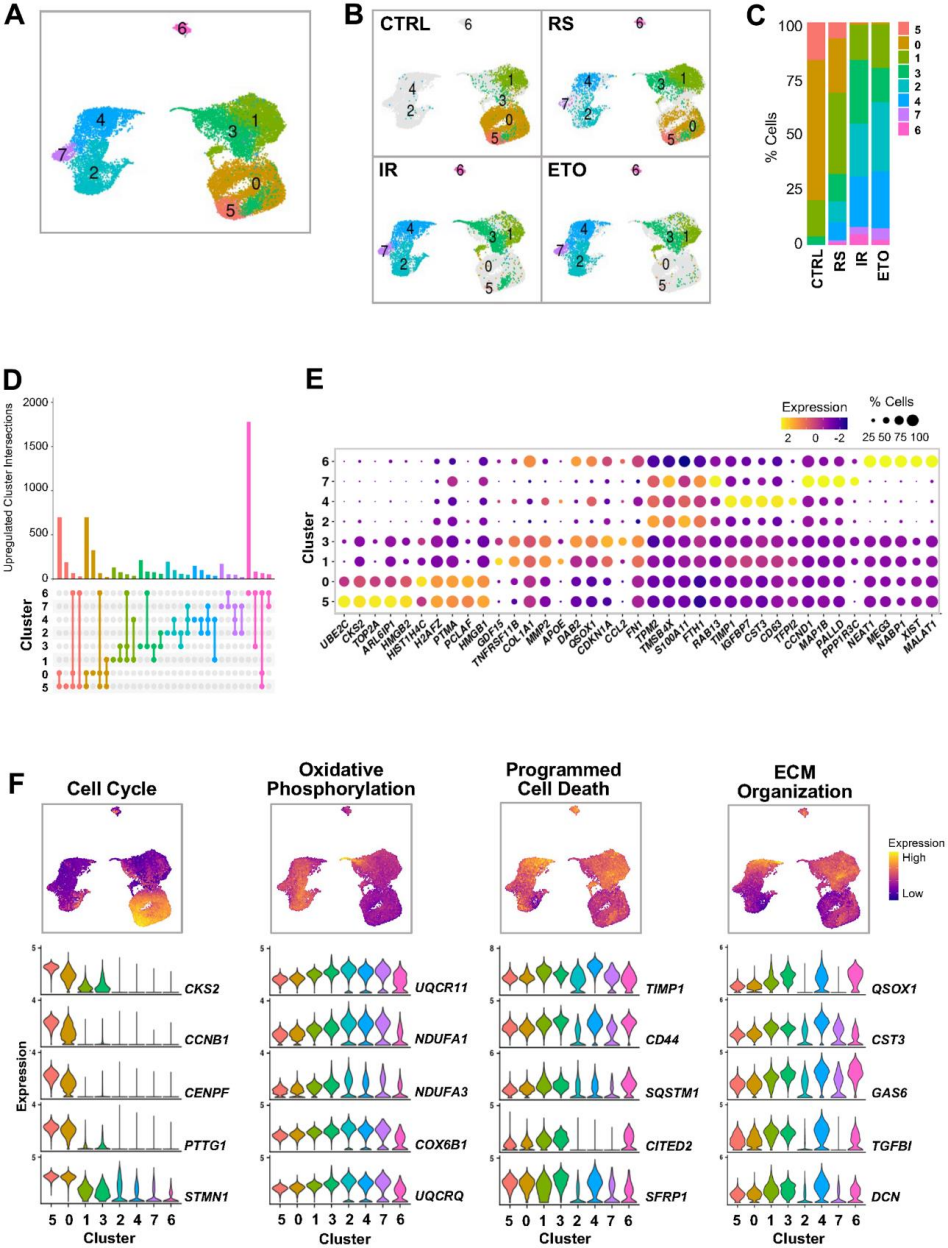
**Clustering analysis of integrated single-cell senescence models.** (**A**) UMAP illustrating the cell clusters with different gene expression profiles, distinguished by unsupervised clustering of integrated samples from Figure 1. (**B**) Distribution of cell clusters from (**A**) in each sample analyzed. (**C**) Percent composition of cell clusters from (**B**) in each sample. (**D**) Overlap of upregulated RNA sets among clusters. Set size: number of upregulated RNAs in each cluster. Each column shows number of genes encoding RNAs that are either unique for one cluster (single dot) or shared by clusters (dots connected by lines). (**E**) Top highest expressed marker RNAs in each cluster. Dot color represents average gene expression levels scaled across all clusters, and dot size indicates percentage of cells expressing specific gene in each cluster. Clusters are ordered by similarity of the transcriptomes in (**A**). (**F**) Select GO terms of GSEA performed for each cluster (Supplementary Figure 1B). Cells are colored by gene expression signature scores of indicated GO terms assessed for each cell and presented in UMAP space (top). Violin plots show expression levels of RNAs from top genes contributing to scoring across all clusters (bottom).

To explore the transcriptomic patterns and their contribution to the senescence models, we performed clustering analysis of the integrated data. The analysis identified eight cell clusters with distinct transcriptomic patterns organized into four major cell groups: clusters 5 and 0 in one group, clusters 1 and 3 in the second, clusters 2, 4, and 7 in the third, and cluster 6 at the greatest distance from other clusters in the UMAP space (Figure 2A). Cells in the CTRL, RS, IR, and ETO showed different distributions across these groups (Figure 2B, 2C and Supplementary Table 3).

The cells of clusters 5 and 0 represented almost 80% of the CTRL cells, 30% of the RS cells, and only ~1% of the IR and ETO cells. They also showed major overlap among them (Figure 2D) with the highest expression of mRNAs encoding proteins that promote cell cycle, proliferation, and transcription, including *CKS2, HMGB1/2, TOP2A, PCLAF, PTMA, CCNB1* and *MKI67* mRNAs (Figure 2E and Supplementary Table 4). Accordingly, the GSEA of GO terms performed on differentially expressed genes in each cluster compared to the rest of the cells, showed positive enrichment of gene sets related to cell cycle and cell division in clusters 5 and 0 (Figure 2F and Supplementary Figure 1B), supporting the proliferative status of these cells.

The cells in clusters 1 and 3 represented the remaining 20% of CTRL, almost 50% of RS cells, and ~40% of each IR and ETO treated populations. These clusters included high abundance of mRNAs encoding proteins associated with growth arrest (e.g., *CDKN1A* and *GDF15* mRNAs) and protein components of the senescence-associated secretory phenotype (SASP) (e.g., *COL1A1*, *MMP2* and *CCL2* mRNAs). Accordingly, GSEA revealed that clusters 1 and 3 included cells with reduced growth and increased secretory activity.

The cells in clusters 2, 4, and 7 were almost devoid (~0.5%) of CTRL cells, but comprised ~20% of the RS population, and > 50% of each of the IR and ETO populations. These clusters shared expression of many top markers *TPM2, TMSB4X, S100A11*, and *FTH1* mRNAs and were highly distinct from other clusters (Figure 2A–2E). Of the three clusters (2, 4, and 7), cluster 2 showed the highest levels of mRNAs encoding oxidative phosphorylation proteins, while cluster 4 appeared to be closely associated to the SASP, with highest expression of mRNAs encoding tissue and extracellular matrix (ECM) remodeling proteins (*TIMP1*, *IGFBP7*, *MMP1*, and *SERPINE2* mRNAs) (Figure 2F and Supplementary Figure 1B and Supplementary Table 4). Cluster 7 cells displayed the most marked upregulation of known senescence markers, including *CDKN2A*, *CCND1*, and *CCND2* mRNAs, as well as *PINK1* mRNA, encoding the protein PINK, which is implicated in Parkinson’s disease and regulates mitochondrial quality control [19], and *IGFBP5* mRNA, encoding a potent inducer of fibrosis and ECM remodeling [20]. Together, the cells in clusters 2, 4, and 7 represent a heterogeneous senescent cell population with transcriptomic patterns reflecting different senescent cell states with distinct phenotypes.

Finally, cluster 6 cells showed the most distinct transcriptomes from all other cells and were present in all senescent cells but not in CTRL cells (Figure 2A–2D). Interestingly, the highest expressed transcripts distinguishing this group included multiple long noncoding RNAs (lncRNAs), such as *NEAT1*, *MALAT1*, *XIST*, and *MEG3* (Figure 2E and Supplementary Table 4). Supporting the senescent character of these cells, this cluster displayed reduced expression of mRNAs encoding proteins with roles in oxidative phosphorylation and cell death, as well as increased expression of mRNAs encoding proteins implicated in ECM organization; however, due to their low number, we were unable to characterize cluster 6 cells in depth (Figure 2E and Supplementary Figure 1B).

In summary, we identified four main groups of cells with different transcriptomic profiles consistent with different functions. Proliferating cells (clusters 5 and 0) mainly represented the CTRL population, growth-arrested cells (clusters 1 and 3) mainly comprised the RS population, and two groups of cells with senescent phenotype (clusters 2, 4, and 7, as well as the lone cluster 6) were enriched in the IR and ETO populations. Cells in the latter two groups (clusters, 2, 4, 6, and 7) expressed low levels of mRNAs encoding cell cycle and cell death proteins, but also expressed heterogeneous senescence features, possibly reflecting a mixture of various senescence forms or cell states in this phenotype.

### Cell state dynamics in ETO-induced senescence initiation and progression

**Figure 3:**
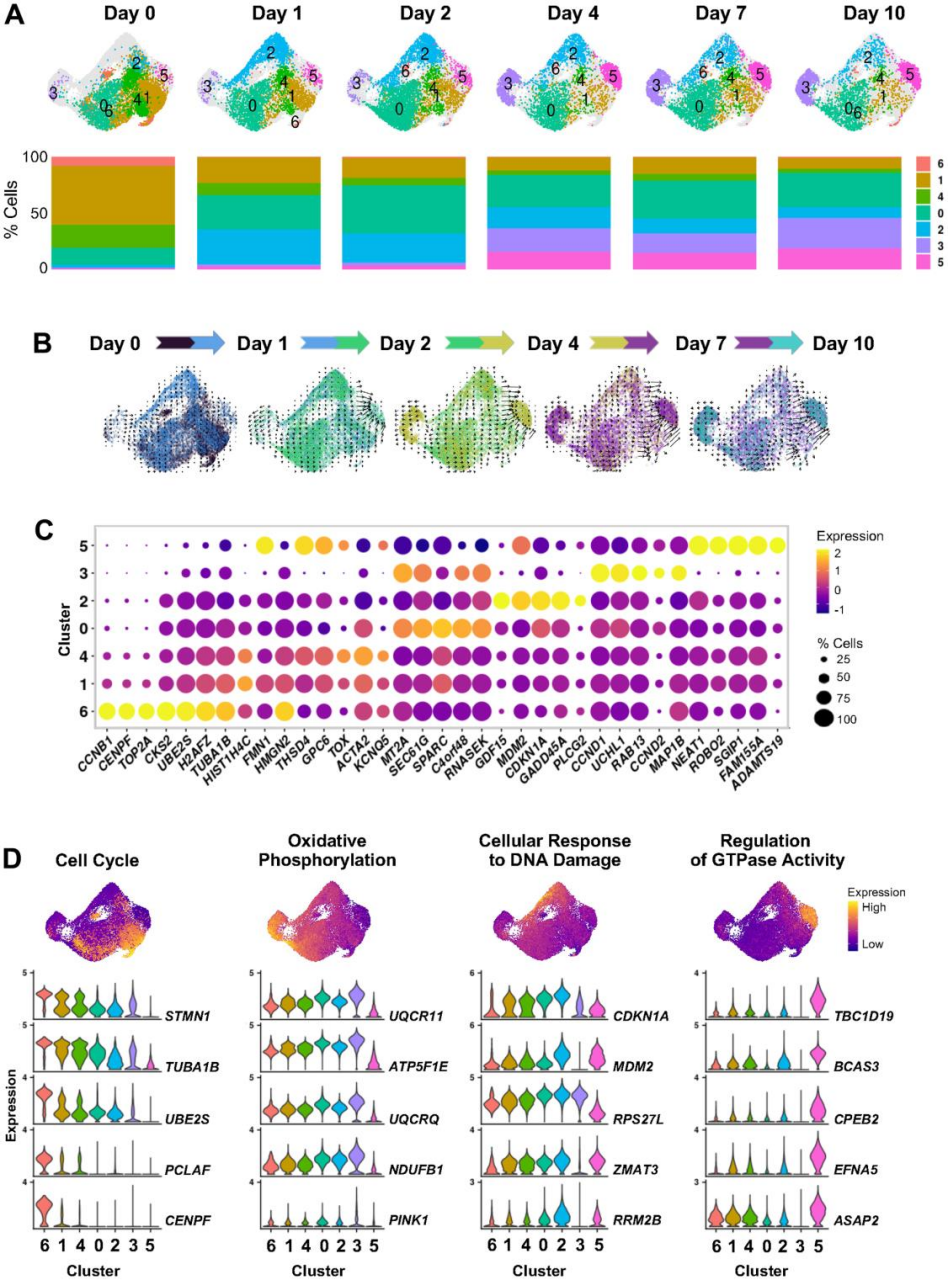
**Cell states evolving over time after ETO-induced senescence.** (**A**) UMAP plots illustrating changes in the distribution of cell clusters identified by scRNA-seq analysis at the indicated time points after beginning treatment with 50 μM ETO added to the culture media (top). Percent composition of cell clusters in each time point during ETO treatment (bottom). (**B**) RNA velocity projected onto the UMAP plot illustrating the direction of transitions among cell clusters. To preserve the temporal variations in trajectories, velocity estimates were calculated in paired contiguous samples. The color dots indicate cells in each pair of time points. Arrow length indicates the estimated rate of transcriptomic changes and arrowhead shows predicted direction of these changes. (**C**) Most highly expressed marker RNAs in each cluster. Dot color represents average RNA expression levels scaled across all clusters and dot size indicates percentage of cells expressing a specific RNA in each cluster. Clusters are ordered by similarity of the transcriptomes of cells in (**A**). (**D**) Select GO terms from Supplementary Figure 1C. Cells are colored by transcriptomic signature scores of indicated GO terms assessed for each cell and presented in UMAP space (top). Violin plots show the expression patterns of top RNAs contributing to scoring across all clusters (bottom).

Second, to systematically investigate how the heterogeneity of cells undergoing senescence unfolds over time, we treated WI-38 fibroblasts with 50 μM ETO and prepared scRNA-seq libraries at 0 (untreated), 1, 2, 4, 7, and 10 days after treatment. Clustering analysis after integrating cells from all time points identified seven clusters with different transcriptomic states that uncovered dynamic changes during senescence initiation and progression (Figure 3A and Supplementary Table 5). Estimation of cell progression over time, as measured by RNA velocity [assessed by comparing spliced relative to unspliced versions of the same transcripts (Materials and Methods)], showed day-to-day changes in directionality and dynamics of cell-state trajectories during the 10-day response to ETO (Figure 3B). To gain insight into the transcriptomic changes contributing to the observed transitions, we examined the transcriptomic profiles of each cell cluster and assessed their predicted functions (Figure 3C, 3D and Supplementary Figure 1C and Supplementary Table 6).

We observed that cells with transcriptomes of clusters 6, 1, and 4 represented over 80% of the untreated population (Day 0), but dramatically decreased to about 30% cells at Day 1 after ETO treatment, and further decreased during the subsequent days (Figure 3A). The cells in these clusters expressed high levels of transcripts encoding proteins that promote cell proliferation, such as *CCNB1, HMGB1/2, TOP2A*, and *TUBA1B/C* mRNAs (Figure 3C and Supplementary Table 6). The transcriptomic profile of clusters 6, 1, and 4 populations, along with its high representation in the untreated group and subsequent reduction after ETO treatment, support various proliferative states of cells in these clusters that were suppressed after stress. In contrast, cluster 0, encompassing around 15% of total cells at Day 0, grew to ~30% cells at Day 1, and stayed at similar abundance through the rest of the time evaluated, indicating that this population does not change drastically by the ETO treatment. Interestingly, the RNA velocity analysis showed multidirectional vectors in clusters 6, 1, 4 and 0, indicating that these cells may be ‘hubs’ of different cell fates after ETO treatment (Figure 3B).

This analysis further uncovered that cluster 2 cells, present at only 2% in the Day 0 population, expanded to over 30% of cells by Day 1 after ETO exposure, then gradually decreased for the remainder of the time course to <10% in Day 10 cells. The brief appearance of the cluster 2 transcriptome at Day 1 after ETO treatment, followed by a time-dependent disappearance of this cluster (Figure 3A), underscored the transient nature of this transcriptome in the cellular response to the initial DNA damage following exposure to ETO. Importantly, cluster 2 cells showed high levels of transcripts encoding proteins that are implicated in p53-mediated stress-response pathway such as *CDKN1A*, *GADD45A*, *GDF15*, and *MDM2* mRNAs (Figure 3C, 3D, and Supplementary Table 6). This initial induction at Day 1 was accompanied by a gradual decrease of the levels of these transcripts at the later time points in the ETO senescence program (Supplementary Figure 2). In addition, GSEA revealed enrichment in cluster 2 of several GO terms associated with the cellular response to DNA damage (Supplementary Figure 1C). In all, our findings indicate that cluster 2 represents a cellular state of early transcriptomic response to DNA damage and support the importance of the p53 pathway at a time of cell fate decisions to undergo senescence.

Finally, clustering uncovered two cell subpopulations that gradually emerged over time, clusters 3 and 5 (Figure 3A, 3B). Each of these clusters represented only 1-3% of Day 0, 1, and 2 populations, then expanded to ~20% (for cluster 3) and 15% (for cluster 5) of the population on Day 4 and beyond. The RNA velocity vectors indicated different trajectories, some distinctly towards cluster 3, and a more intense trajectory towards cluster 5, suggesting the existence of divergent cell transitions and cell fate developments towards these two subpopulations. Cluster 3 was enriched in membrane and oxidative phosphorylation GO gene sets and exhibited high expression of *CCND1*, *CCND2*, *UCHL1*, and *CDKN2A* mRNAs, encoding known senescence proteins (Figure 3C, 3D and Supplementary Table 6). On the other hand, cluster 5 showed decreased oxidative phosphorylation and upregulation of GTPase activity GO gene sets. Interestingly, cluster 5 expressed high levels of many lncRNAs, as it was observed for cluster 6 in the aggregate senescence models (Figure 2E), underscoring the transcriptomic similarity of these two clusters. These findings indicate that cells in clusters 3 and 5 may represent different forms of the senescent phenotype emerging via distinct cell fate programs in response to cellular stress. In summary, the transcriptomic analysis performed for each cluster offered details and validation of the dynamic transitions of transcriptomically distinct cell states during ETO-induced senescence initiation and progression.

### Cell proliferative status influence gene response to ETO treatment and cell fate trajectory

**Figure 4:**
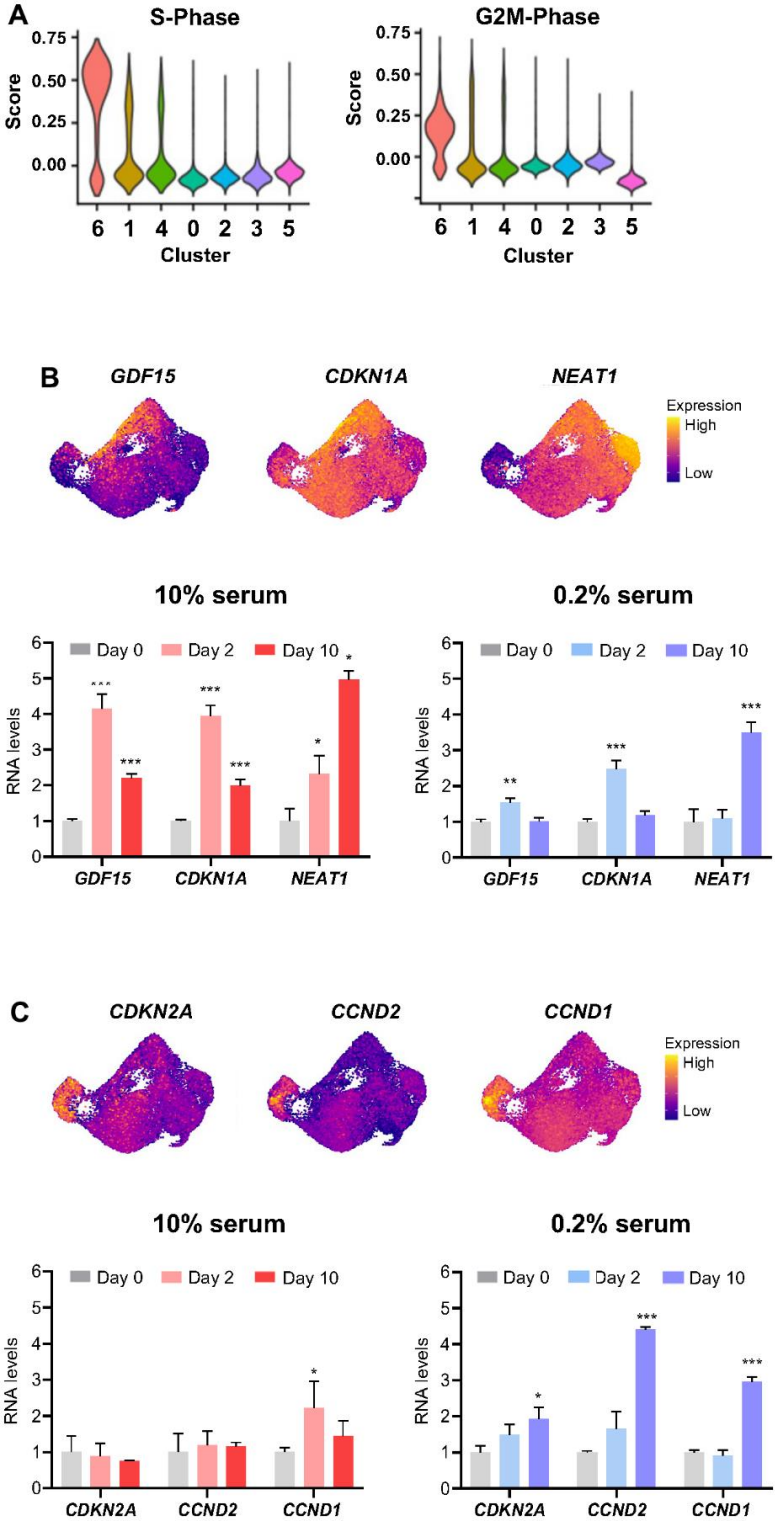
**Influence of cell proliferative status on transcriptomic response to ETO treatment.** (**A**) Cell cycle states of ETO time course cell clusters determined by the ‘CellCycleScoring’ function in Seurat. (**B**, **C**) Relative levels of select RNAs upregulated in cluster 2 (**B**), and cluster 3 (**C**) compared to the rest of the cells (top). The expression levels of RNAs shown (top) after ETO treatment (Day 2, Day 10) in cells cultured in 10% or 0.2% serum media were quantified by RT-qPCR analysis (bottom). Relative RNA levels were normalized to *GAPDH* mRNA levels and compared to untreated cells (Day 0). Significance was assessed by two-tailed unpaired Student’s *t*-test, n = 3, *p < 0.05, **p < 0.01, ***p < 0.001.

Given that the cell cycle stage may influence the cellular response to stress conditions, we asked if the cell cycle phase of the abundant clusters (6, 1, 4, and 0) at Day 0 (untreated) might inform us about different responses to ETO treatment. To check this possibility, we assigned a cell cycle phase score to individual ETO time course cells by using ‘CellCycleScoring’ function in Seurat. We found that the majority of cells from cluster 6 and many cells from clusters 1 and 4 exhibited high scores for S and G2/M phase (Figure 4A), supporting the proliferative state of these populations which were more abundant on Day 0 but were significantly diminished on Day 1 after ETO treatment; instead, on Day 1, we observed the appearance of cluster 2 transcriptome, with upregulation of DNA damage response (DDR) mRNAs (Figure 3). Cell cycle phase scores further confirmed that cluster 0 cells were growth arrested and only changed slightly with exposure to ETO (Figure 4A). Remarkably, RNA velocity vectors after ETO exposure (Figure 3B) showed that cluster 0, representing growth-arrested cells, progressed toward cluster 3, while (proliferative) cells in clusters 6, 1, and 4 were directed towards cluster 5. Thus, we postulated that in response to ETO, the proliferating cells of clusters 6, 1 and 4 shifted their gene expression patterns toward formation of the DDR population of cluster 2, and later progressed to cluster 5, adopting a senescence transcriptome. On the other hand, cell cycle-arrested, cluster 0 population perhaps responded to DNA damage only marginally, and further transitioned to adopt the transcriptome of senescent cells in cluster 3.

To distinguish between these possibilities, we treated populations that were predominantly proliferating (cultured in 10% serum) and predominantly growth-arrested (cultured in 0.2% serum) with 50 μM of ETO; 48 h and 10 days later, we measured the expression levels of selected RNAs by RT-qPCR analysis. We found that proliferating cells (10% serum) exposed to ETO expressed high levels of DDR RNAs (markers of cluster 2), while arrested cells (0.2% serum) showed only slight changes in these RNAs at 48 h after ETO treatment (Figure 4B). Conversely, the senescence markers of cluster 3 were robustly upregulated by day 10 when cells were arrested (0.2% serum) at the time of ETO treatment, but were unchanged if the cells were proliferating at the time of ETO treatment (Figure 4C). Collectively, these results indicate that the proliferation status at the time of senescence-triggering damage affects gene expression patterns and determines subsequent trajectories of cell fate during senescence progression.

### scRNA-seq analysis identified two distinct clusters of senescent cells

**Figure 5:**
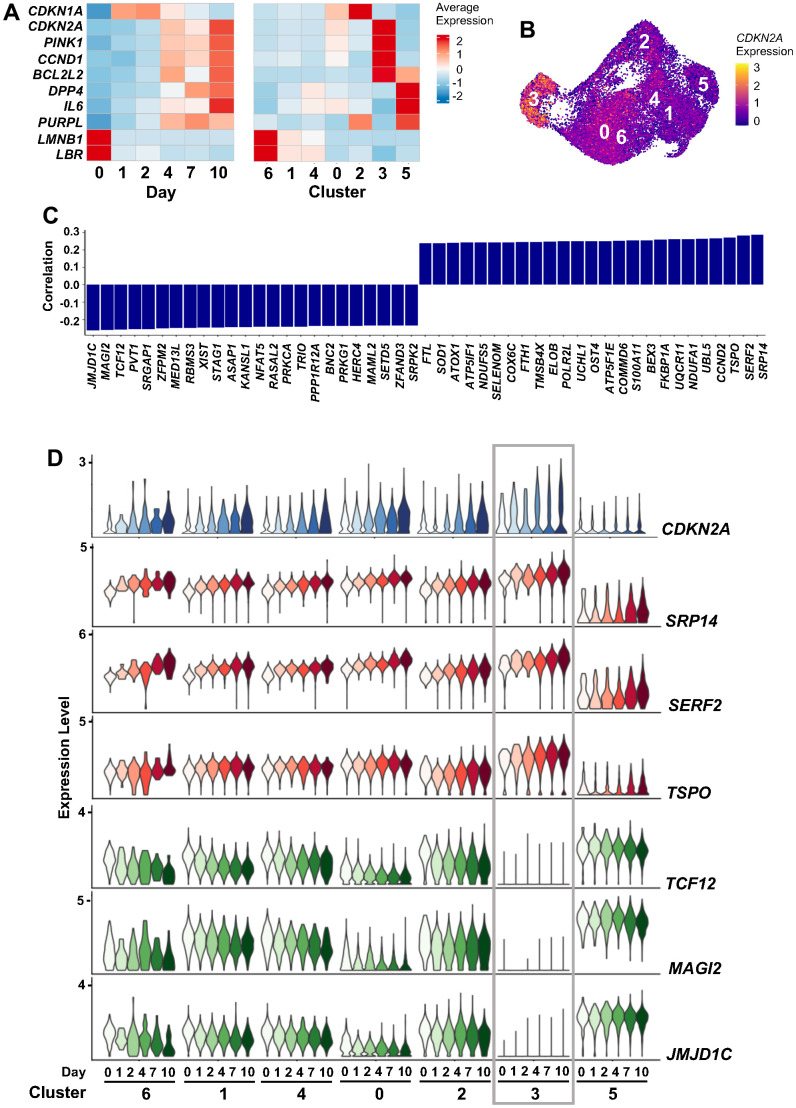
***CDKN2A* mRNA expression during ETO treatment.** (**A**) Heatmaps showing changes in the expression levels of select senescence-associated transcripts across 10 days of ETO exposure (left), and the contribution of each cell cluster to these changes (right). (**B**) Feature plot showing increased *CDKN2A* mRNA expression in cluster 3. (**C**) Top RNAs correlated with *CDKN2A* mRNA expression levels by Pearson correlation coefficient; significance was calculated using paired student’s *t*-test, p < 0.01. (**D**) Expression of *CDKN2A* mRNA and its top three positively and negatively correlating RNAs from (**C**). Changes in RNA levels in each cluster and time point are indicated.

Our study revealed the progressive rise of two transcriptomically distinct cell subpopulations (clusters 3 and 5) during ETO-induced senescence (Figure 3A, 3B). To ensure that these cells represented a senescent state, we evaluated the expression levels of genes commonly associated with different hallmarks of cellular senescence [5, 21] (Figure 5A, left heatmap). We confirmed ETO-induced upregulation of *CDKN1A* and *CDKN2A* mRNAs (encoding the growth arrest proteins p21 and p16), *BCL2L2* mRNA (encoding BCL2L2, a protein that promotes cell survival)*, IL6* mRNA (encoding the SASP factor IL6), *PINK1* mRNA (encoding protein PINK1, which promotes mitochondrial homeostasis), *CCND1* mRNA (encoding CCND1/cyclin D1, required for the G1/S transition), *DPP4* mRNA (encoding the protease DPP4), and *PURPL* (a lncRNA that modulates p53 signaling); we also confirmed ETO-induced declines in the levels of *LMNB1* and *LBR* mRNAs, encoding proteins with key functions in chromatin organization near the nuclear envelope [4, 22–24]. Furthermore, we validated changes in the transcriptome previously reported by bulk RNA-seq analysis [25] (Supplementary Figure 3, left heatmap). Importantly, we found that cluster 3 and cluster 5 cells were the main drivers of the senescence-related changes in the expression of these RNAs (Figure 5A, right heatmap; Supplementary Figure 3, right heatmap). For instance, the total increase in expression levels of *CDKN2A*, *CCND1*, and *PINK1* mRNAs was mainly the result of their elevation in cluster 3 cells, the increase in levels of *DPP4* mRNA, *IL6* mRNA, and *PURPL* was due to changes in cluster 5, and the pro-survival *BCL2L2* mRNA was elevated in cells of both clusters 3 and 5. Interestingly, the transcriptome of cluster 3 was similar to those of clusters 2, 4, and 7 from the senescence models dataset, while the transcriptome of cluster 5 was similar to that of cluster 6 from the senescence models dataset (Supplementary Figure 4A, 4B). Taken together, these findings support the existence of transcriptomically distinct populations of senescent cells in WI-38 fibroblast senescence models.

As one of the most referenced markers for senescence, we considered the high expression of *CDKN2A* mRNA in cluster 3 (Figure 5B) important for a deeper understanding of the heterogeneity of senescent cells. Analysis of the correlation of *CDKN2A* mRNA levels with the levels of other genes across all cells revealed positive correlation with multiple mRNAs encoding oxidative phosphorylation-related proteins (e.g., *NDUFA1*, *UQCR11*, *COX6C*, and *NDUFS5* mRNAs) (Figure 5C). We also found time-dependent changes in *CDKN2A* mRNA and other transcripts that increased (*SRP14*, *SERF2*, and *TSPO* mRNAs) or decreased (*TCF12*, *MAG12*, and *JMJD1C* mRNAs) over time, reflecting a dynamic continuum of changes following ETO exposure (Figure 5D). The high similarity in gene expression patterns between clusters 3 and 0 recapitulates the similar transcriptomes of these two clusters seen earlier (Figure 3C, 3D), further solidifying the possibility of transitions between cells in clusters 0 and 3. However, the predominant accumulation of cells with high *CDKN2A* mRNA expression in cluster 3, accompanied by specific transcriptomic trajectories culminating in cluster 3 cells revealed by RNA velocity estimation (Figure 3B), suggests that this is a distinct subpopulation.

**Figure 6:**
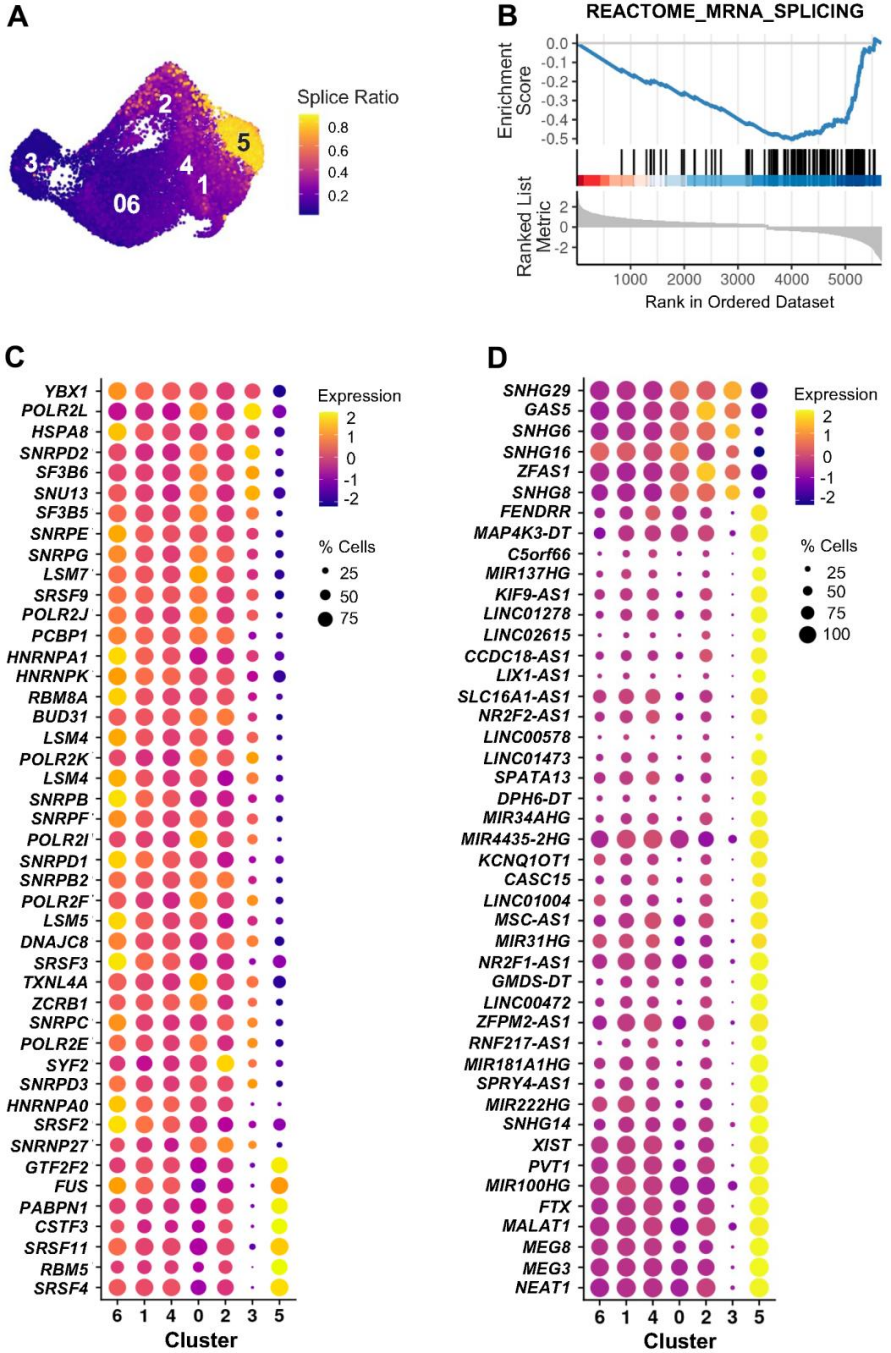
**RNA splicing and lncRNAs in cluster 5 of ETO time course.** (**A**) Splice ratio illustrating increased unspliced RNAs in cluster 5. Splice ratio for individual cells was calculated as the number of unspliced transcripts relative to total RNA content. (**B**) GSEA enrichment plot of “REACTOME_MRNA_SPLICING” gene set in cluster 5 in comparison to all other cells. Normalized enrichment score NES = -2.42, adjusted p-value < 0.001. (**C**) Top 45 mRNAs encoding splicing-associated proteins identified by GSEA in (**B**) that showed greatest difference in expression between cluster 5 and all other cells. (**D**) Top 45 lncRNAs showing greatest changes of expression in cluster 5 compared to the rest of cells. In (**C**, **D**) dot color represents average RNA levels scaled across all clusters and dot size indicates percentage of cells expressing the specific RNAs in each cluster.

Cluster 5, contrary to cluster 3, showed low and unaltered levels of *CDKN2A* mRNA and *CDKN2A*-correlated genes over time after ETO exposure (Figure 5D). Instead, an interesting feature of the transcriptome in cluster 5 became apparent by analyzing RNA velocity, which distinguished pre-mature (unspliced) from mature (spliced) RNAs. Calculation of the splice ratio for each cell (Materials and Methods) showed increased unspliced RNAs in cluster 5 cells when compared to the rest of the cells (Figure 6A and Supplementary Figures 5A, 6A). It is noteworthy that, similarly to cluster 5, cluster 6 in the combined analysis of senescence models (Figure 2) also exhibited increased unspliced RNA levels (Supplementary Figures 5B, 6B, 6C), confirming the reproducibility of this feature through different approaches. To search for factors that could influence decreased RNA splicing in cluster 5 cells, we performed GSEA of the “REACTOME_MRNA_SPLICING”, a set of genes encoding proteins that regulate RNA splicing. The discovery of a negative enrichment score of this gene set in cluster 5 cells suggested that these cells expressed reduced levels of splicing factors compared to all other clusters (Figure 6B). As shown in Figure 6C, while most of these mRNAs encoding RNA splicing proteins were widely expressed across manifold clusters, they were strongly downregulated in cluster 5 (Supplementary Table 7), perhaps affecting the RNA processing events specifically in this cluster. Interestingly, many lncRNAs appeared as top markers distinguishing cluster 5 from other clusters (Figure 3C and Supplementary Table 6). Given the emerging role of lncRNAs as important modulators of gene expression in multiple cellular processes, including senescence, we evaluated changes in expression of lncRNAs across all clusters, finding again that most of them were selectively elevated in cluster 5 (Figure 6D and Supplementary Table 8). In sum, we found two distinct subpopulations of senescent cells (Supplementary Figure 7): cluster 3 cells express high levels of the traditional senescence marker *CDKN2A* and cluster 5 cells show dysregulated RNA splicing and high expression of multiple lncRNAs.

## DISCUSSION

The process of senescence involves diverse cell states that evolve dynamically in response to sublethal damage and enable highly diverse transcriptomic and phenotypic responses [26]. The existence of a ‘division of labor’ within a senescent population was hypothesized, whereby different senescence traits were elicited by subsets of senescent cells. To increase our understanding of the cell populations carrying out this heterogeneous this response, we performed scRNA-seq analysis of WI-38 human diploid fibroblasts on two levels: first, we studied side-by-side the senescent phenotype achieved following exposure to different triggers (RS, ETO, IR) and, second, we analyzed the progression of the senescent phenotype over time in the ETO model.

In the first analysis, we found that senescent cells following RS displayed wide heterogeneity, while cells rendered senescent by IR or ETO showed gene expression patterns comparable to one another (Figures 1B, 2B). Unsupervised clustering analysis of integrated data distinguished four main functionally different groups of cells with altered representation in each senescent model. Proliferating cells (clusters 5 and 0) mostly (80%) comprised CTRL cells, but also comprised 30% of RS cells, and were negligible in IR and ETO cells. Growth-arrested cells without overt senescence features (clusters 1 and 3) comprised 20% of CTRL cells, almost 50% of RS cells, and 40% of IR or ETO cells. Cells displaying classical senescence markers (clusters 2, 4, and 7), expressing increased levels of mRNAs encoding p21, oxidative phosphorylation proteins, SASP factors, ECM proteases, and proteins that inhibit apoptosis, were absent from CTRL cells, contained 20% of RS cells, and represented more >50% of IR and ETO cells. Finally, a distinct senescent population (cluster 6) was identified with different features of cell senescence, such as increased levels of mRNAs encoding ECM organization proteins and antiapoptotic proteins, low levels of mRNAs encoding oxidative phosphorylation proteins, and high levels of lncRNAs; these cells were found in all senescence models but not in the CTRL population. The above results reveal a broad transcriptomic diversity from different senescent paradigms, with the unexpected discovery that many populations have cell states represented in clusters 2, 4, and 7. These findings agree with a growing number of studies recognizing the highly diversified nature and gene expression signatures of senescent cells [2, 5, 23, 27].

In the second analysis, we systematically investigated the changes in cell transcriptomes after senescence initiation and progression by performing kinetic scRNA-seq analysis of ETO-induced senescence. This approach led us to characterize the evolution of different cell states over time as cells acquired a senescent phenotype (Figure 3). The various proliferative states of cells observed in clusters 6 (highly proliferating), 1, and 4 (slower proliferating) represented 80% of Day 0 (untreated) cells, then rapidly diminished in 24 h (Day 1) after ETO treatment. Instead, on Day 1, we observed the appearance of cluster 2 cells which gradually declined after Day 2. Cells of cluster 2 expressed high levels of DDR genes, indicating that DNA damage induced transcriptomic changes in this cell cluster in response to ETO treatment. In contrast, the growth-arrested cells of cluster 0 only slightly increased in number by Day 1 and remained unchanged for the remainder of the time studied. Finally, on Day 4, we found a robust increase in two transcriptomically distinct cell populations (clusters 3 and 5; Figure 3A, 3B), suggesting that they follow different functional paths in the senescence program. The progressive emergence of cells in these clusters, RNA velocity, and transcriptomic profiles indicate that specific cells are recruited to each of these senescent clusters. RNA velocity analysis indicated that the senescent cells of cluster 3 likely originate from the non-proliferative cell cluster 0. Notably, cluster 3 cells exhibited high levels of the canonical senescence marker *CDKN2A* mRNA (Figure 5B); the encoded protein, p16 increases in the vast majority of senescent cells and is considered to be essential for maintenance of a senescent state [28]. However, not all senescent cells express high levels of p16, and conversely, p16 can be upregulated also in arrested non-senescent cells [2, 29]; therefore, increased p16 expression may be a helpful marker of senescence but on its own it is not sufficient to identify a senescent cell [30, 31].

The time course analysis further revealed that cluster 5 senescent cells exhibited an accumulation of unspliced transcripts along with dysregulated expression of genes encoding proteins implicated in RNA splicing (Figure 6), highlighting an impairment in RNA splicing events in this population. High amounts of unspliced RNAs and increased RNA dynamics were previously reported in aged mouse cardiac fibroblasts [32], and disrupted balance of RNA splicing and changes in expression of regulatory splicing factors are considered to be major contributors to cell senescence and aging [33–36]. The direction of changes in RNA velocity (Figure 3B) led us to propose that after ETO treatment, proliferative cells acquired a transient DDR state (cluster 2) and then transitioned to cluster 5. Therefore, we hypothesized that aberrant splicing could be part of the response to ETO-induced DNA damage. This possibility is supported by studies showing the impact of DNA damage on pathways that control splicing in p53-mediated cellular senescence [15, 37, 38]; however, the mechanisms whereby DNA damage regulates splicing in senescence await more study. Remarkably, cluster 5 cells also expressed high levels of many lncRNAs (Figure 6D), a class of noncoding RNAs that play regulatory roles at multiple gene expression levels, including chromatin remodeling, transcription, mRNA splicing, translation, and post-translational modifications [39]. Although some lncRNAs have been shown to participate in the regulation of alternative splicing (e.g., *NEAT1*, *MALAT1*), cell cycle (*MALAT1*), and senescence (*PURPL*, *MIR31HG*, and *LncRNA-OIS1*) [25, 40–44], the functions of most lncRNAs remain unknown. On the other hand, a growing number of studies indicates that one of the principal mechanisms by which lncRNAs regulate splicing is by interacting with splicing factors to repress or enhance their expression and function [45]. A deeper understanding of a role of lncRNAs in controlling splicing factor function awaits further investigation.

Interestingly, as senescence advanced, we observed increased levels of mRNAs encoding oxidative phosphorylation proteins in cluster 3 cells and reduced in cluster 5 cells (Figure 3D and Supplementary Figure 1C). This result agrees with evidence that senescent cells display increased oxidative phosphorylation while at the same time, they are characterized by mitochondrial dysfunction [46–50]. Although the metabolic changes experienced by senescent cells are poorly understood, the upregulation of oxidative phosphorylation in cluster 3 may point to senescent cells striving to maintain energy production by mitochondria. We also found that the genes involved in regulation of GTPase activity were reduced in cluster 3 and increased in cluster 5; these genes are implicated in membrane trafficking, intracellular vesicle transport, autophagy, and secretory pathways [51]. In all, cluster 3 senescent cells demonstrate a transcriptome consistent with growth arrest (elevated *CDKN2A* mRNA, leading to increased p16/pRB activity), while cluster 5 cells show features of DNA damage-induced p53/p21-mediated cellular senescence (developed through cluster 2, which displays high levels of *CDKN1A* mRNA). The contribution of these two senescence programs was confirmed by analyzing subsets of senescence-related transcripts identified previously via bulk RNA-seq analysis [25]. In addition, we found that the cell cycle status at the time of senescence initiation influenced the transcriptomes of senescent cells (Figure 4). Both p16/pRB and p53/p21 are broadly recognized as central pathways involved in regulating cell cycle arrest and senescence, although the full regulatory networks are complex and not fully understood [52–55]. A limitation of this study is that all analyses were performed in one cell line, human WI-38 fibroblasts. Given that senescence programs can differ depending on cell type, proliferation state, or type of damage, future research in other cell types and in response to other stimuli is warranted.

In summary, single-cell transcriptomic analysis has allowed us to identify the specific populations and the dynamic transition states during senescence initiation and progression. Our data revealed divergent cell state trajectories that resulted in the formation of two distinct forms of senescent cells: cluster 3 cells, which emerged from growth-arrested cells, were characterized by high expression of *CDKN2A* mRNA and mRNAs encoding oxidative phosphorylation proteins, and decreased GTPase activity; and cluster 5 cells, which evolved from proliferative cells that underwent strong ETO-induced DDR, and progressed into cells with reduced oxidative phosphorylation, increased GTPase activity, aberrant RNA splicing, and increased expression of lncRNAs. The transcriptomic details of these two traits may guide future efforts to track senescence markers and inform on cell state transitions during the implementation of senescence. We propose that this study complements ongoing work of senescence in organs *in vivo* and can help design increasingly precise therapeutic interventions aimed at select senescent cell populations.

## Supplementary Material

Supplementary Figures

Supplementary Table 1

Supplementary Tables 2 and 3

Supplementary Table 4

Supplementary Table 5

Supplementary Table 6

Supplementary Table 7

Supplementary Table 8
