# Supplementary material for: Single-cell transcriptomic analysis uncovers diverse and dynamic senescent cell populations
Source: Aging (Albany NY). 2023 Apr 19;15(8):2824–51. doi: 10.18632/aging.204666 (PMC10188353; doi:10.18632/aging.204666)
Supplement: Supplementary Figures [file aging-15-204666-s001.pdf]

SUPPLEMENTARY FIGURES

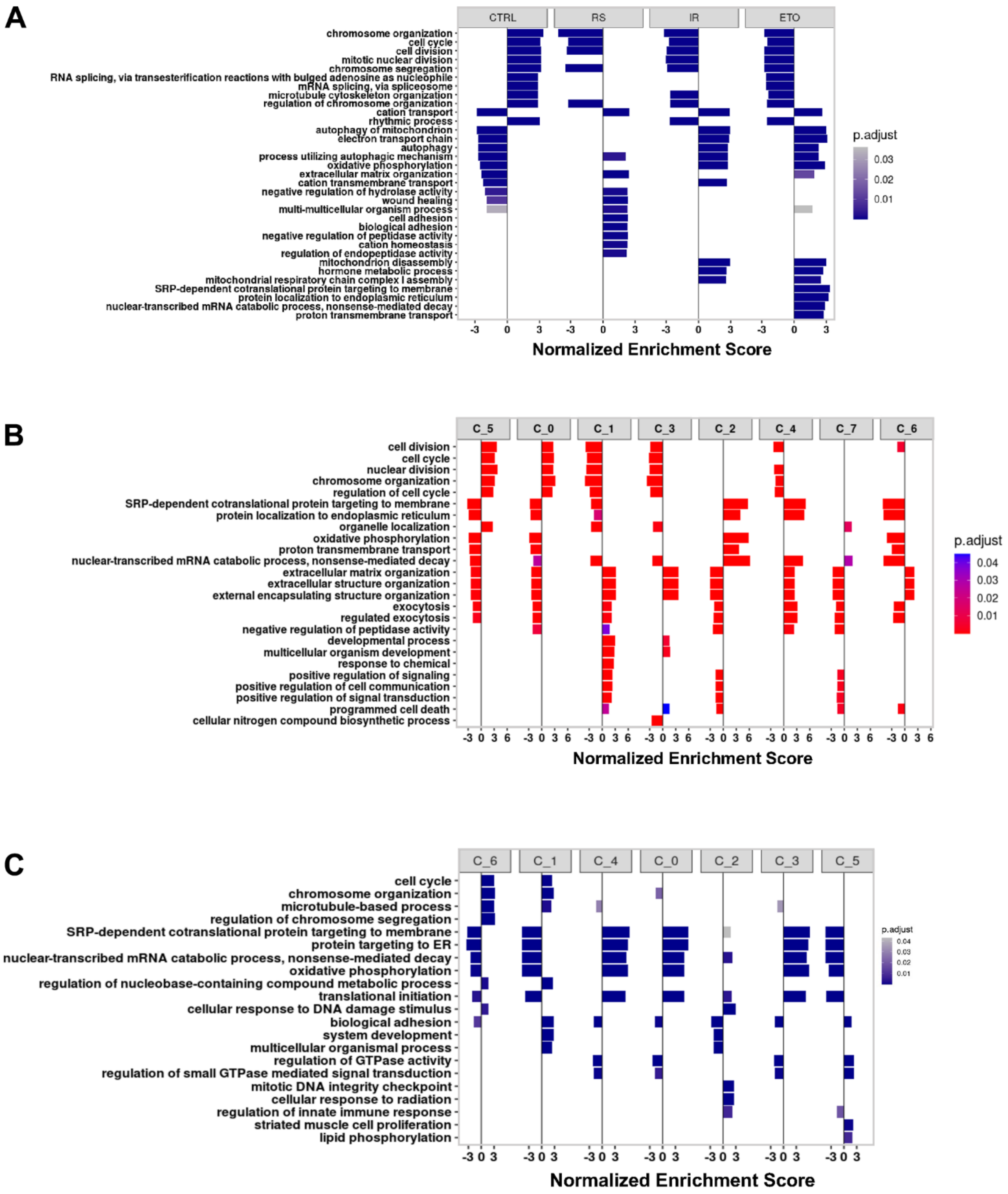

Supplementary Figure 1. Functional characterization of different senescence models. (**A**–**C**) Cell populations CTRL (control proliferating cells, PDL 24), RS (replicative senescent cells, PDL 57), IR (ionizing radiation-treated cells, 10 Gy, 10 days), ETO (etoposide-treated cells, 50 µM, 10 days) were assessed by scRNA-seq analysis. The terms with the highest normalized enrichment score (NES) from the GSEA of GO gene sets obtained using each sample gene markers from Supplementary Table 1 (**A**), each cluster gene markers from Supplementary Table 4 (**B**), and each cluster gene markers from Supplementary Table 6 (**C**).

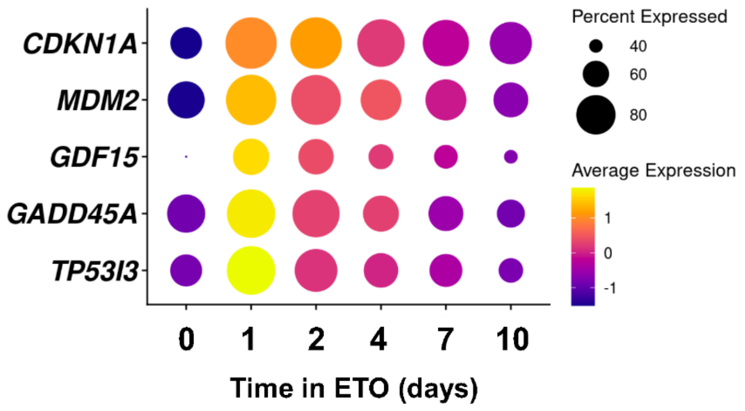

Supplementary Figure 2. Changes in expression of top marker RNAs of cluster 2 at each time point after inducing senescence with ETO.

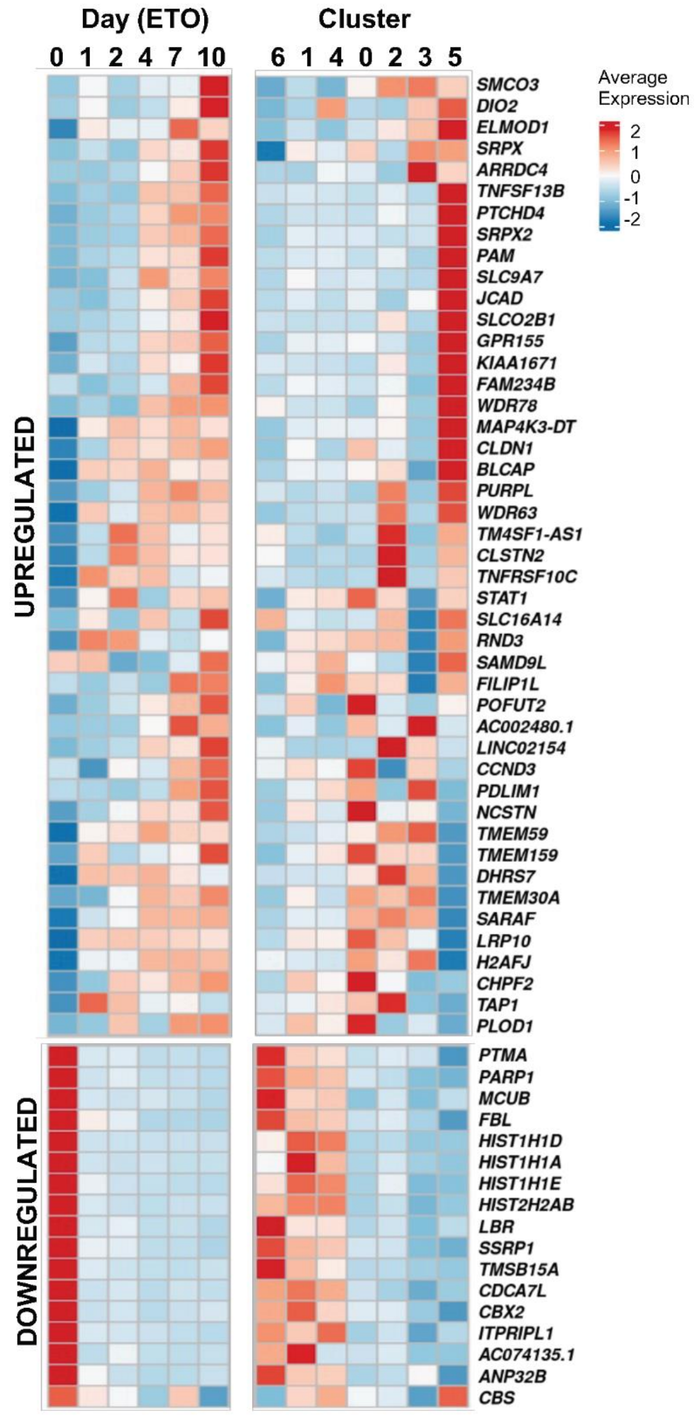

Supplementary Figure 3. Comparison of the senescence-associated transcriptome identified by Casella et al. to the ETO-treated time course in the current study. Heatmaps show changes in the levels of transcripts over 10 days of ETO exposure (left), and the contribution of each cell cluster to these changes (right).

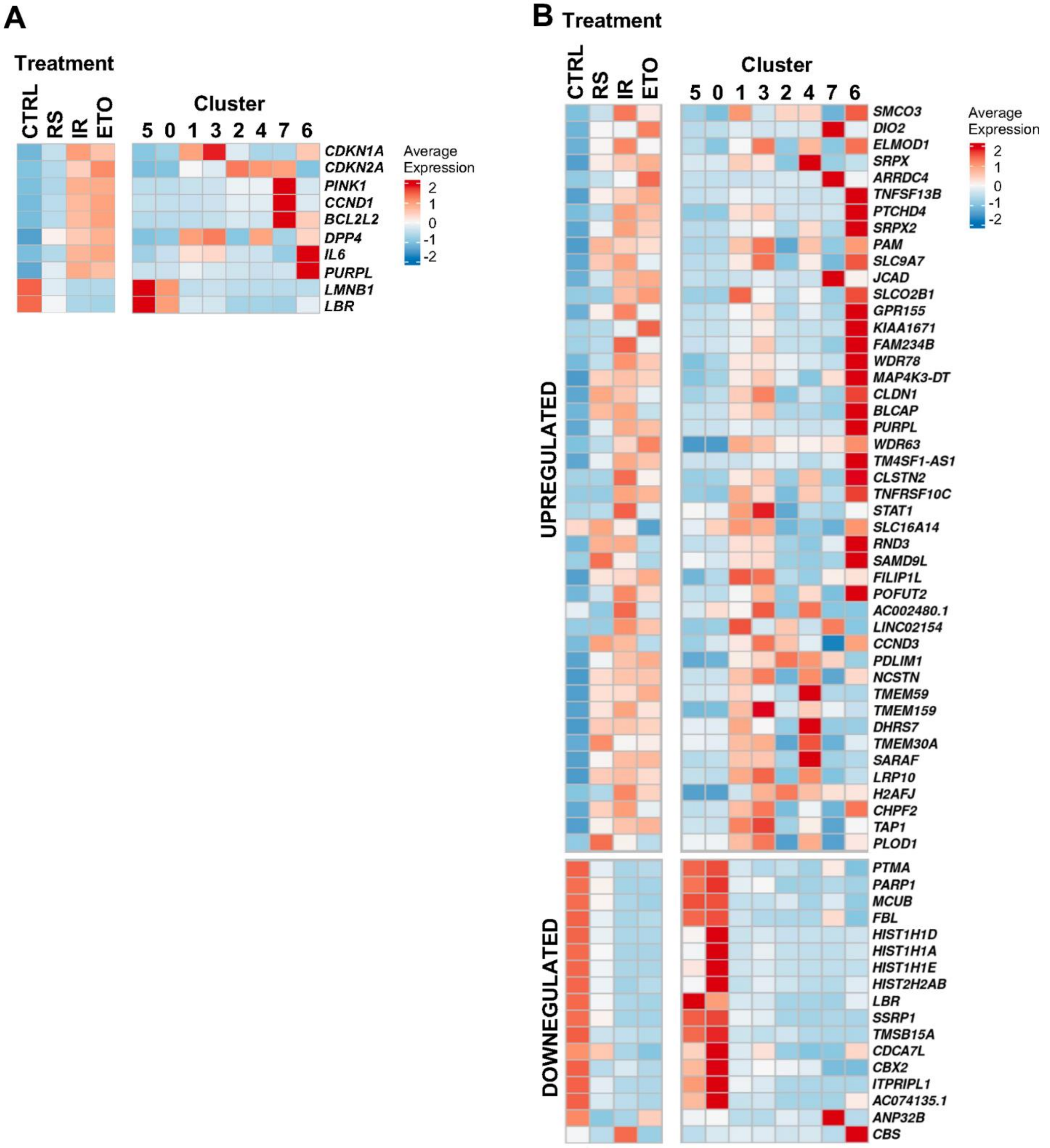

Supplementary Figure 4. Expression of the senescence-associated transcriptome in the senescence models dataset of current study. (**A**) Heatmaps showing changes in expression of common senescence-related RNAs (left), and contribution of each cell cluster to these changes (right). (**B**) Heatmaps showing changes in the expression of genes as described in Supplementary Figure 3.

A

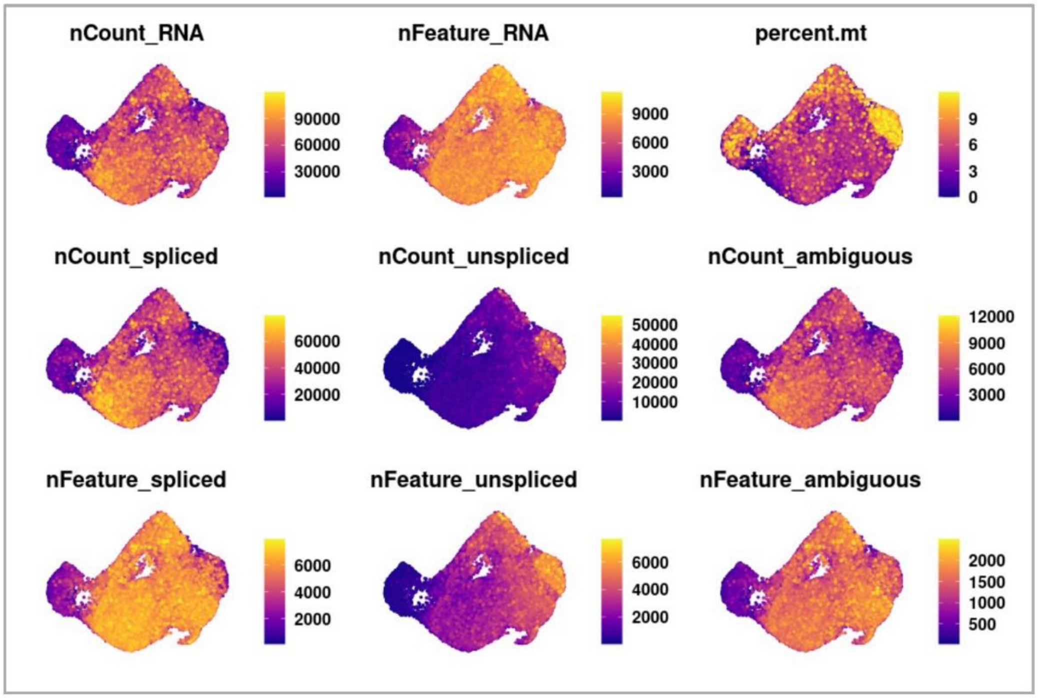

B

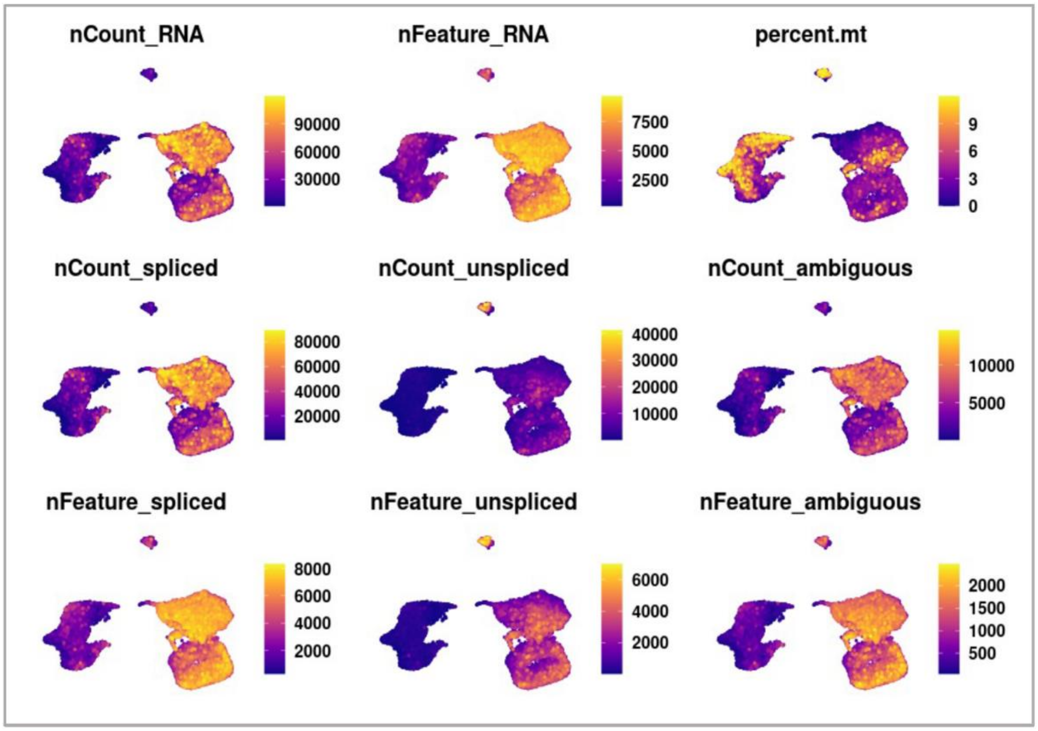

Supplementary Figure 5. Feature plots visualizing the distribution of gene number and counts of datasets in this study. (**A**) ETO time course. (**B**) Senescence models.

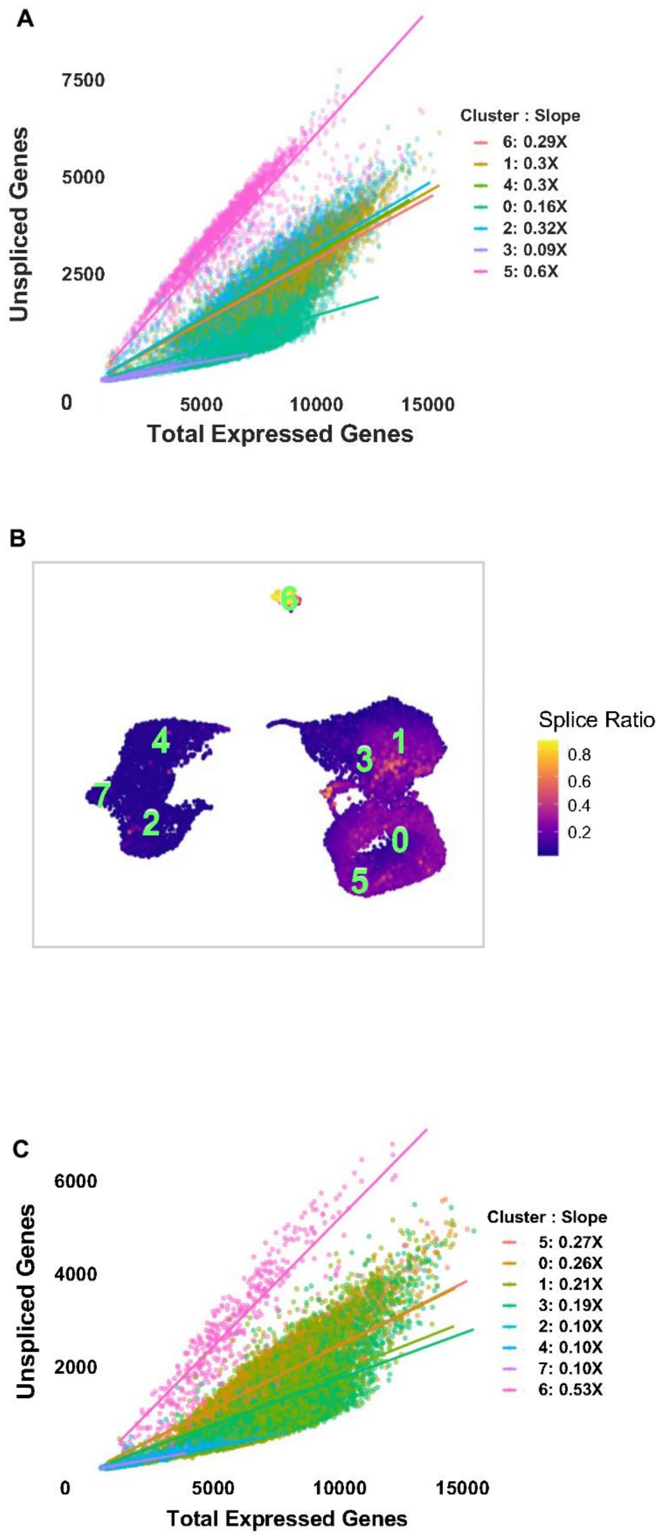

Supplementary Figure 6. Splice ratio in clusters distinguished in senescence models and ETO time-course datasets. (**A**) Number of unspliced RNAs detected (y-axis) versus total number of expressed RNAs (x-axis) in individual cells in the ETO time-course dataset. Linear regression intercepts at 0. The slope of regression model of each cluster is indicated by the cluster in the legend. (**B**) Splice ratios illustrating increased unspliced RNAs in cluster 6 of the senescence models dataset. Splice ratio in individual cells was calculated as the number of unspliced transcripts relative to total expressed RNAs. (**C**) Number of unspliced RNAs detected (y-axis) versus total number of genes expressed (x-axis) in individual cell of senescence models dataset. Linear regression intercepts at 0. Slope of each cluster's regression model is beside the cluster in the legend.

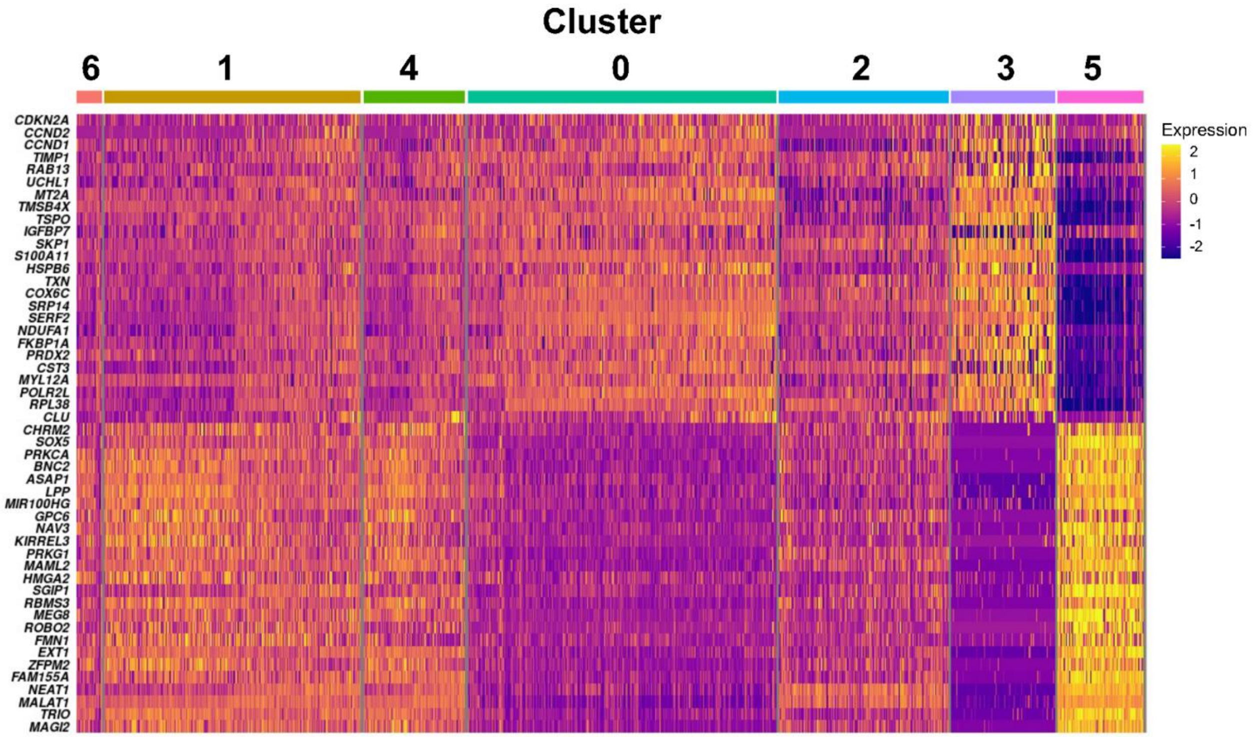

Supplementary Figure 7. Expression of genes discriminating clusters 3 and 5 cells.
