# Supplementary material for: Single-cell transcriptomic analysis uncovers diverse and dynamic senescent cell populations
Source: Aging (Albany NY). 2023 Apr 19;15(8):2824–51. doi: 10.18632/aging.204666 (PMC10188353; doi:10.18632/aging.204666)
Supplement: Supplementary Tables 2 and 3 [file aging-15-204666-s003.pdf]

Supplementary Table 2. List of oligo sequences used in the study.

Transcript name	Primer	Oligo sequence
ACTA2	Forward	AGCCAAGCACTGTCAGGAAT
ACTA2	Reverse	TTGTCACACACCAAGGCAGT
CALD1	Forward	CTGTTCCTGCTGAAGGTGTACG
CALD1	Reverse	CCTACCTTCAAGCCAGCAGTTTC
CCL2	Forward	AGAATCACCAGCAGCAAGTGTCC
CCL2	Reverse	TCCTGAACCCACTTCTGCTTGG
CCND1	Forward	TCTACACCGACAACTCCATCCG
CCND1	Reverse	TCTGGCATTTTGGAGAGGAAGTG
CCND2	Forward	GAGAAGCTGTCTCTGATCCGCA
CCND2	Reverse	CTTCCAGTTGCGATCATCGACG
CDKN1A	Forward	CCTGCCCAAGCTCTACCTT
CDKN1A	Reverse	AAGGCAGAAGATGTAGAGC
CDKN2A	Forward	CGGTCGGAGGCCGATCCAG
CDKN2A	Reverse	GCGCCGTGGAGCAGCAGCAGCT
CENPF	Forward	GCATTGCCATTCCTCTACTGC
CENPF	Reverse	ACCCACATACAAACAGAGATTGTG
CKS2	Forward	TTTTGTCTGCTGCGCCCG
CKS2	Reverse	ACATAACATGCCGGTACTCGT
CYBA	Forward	GCTCATCTGTCTGCTGGAGTATC
CYBA	Reverse	CGGACGTAGTAATTCCTGGTGAG
FTH1	Forward	GACGTTCTTCGCCGAGAGT
FTH1	Reverse	GAACGAGCGCCGGGTT
FTL	Forward	CGGGTCTGTCTCTTGCTTCA
FTL	Reverse	GGAGATGGCCGAGAAGATGG
GAPDH	Forward	CTCTGCTCCTCCTGTTCGAC
GAPDH	Reverse	ACGACCAAATCCGTTGACTC
GAS6	Forward	GGAACTGGCTGAACGGAGAA
GAS6	Reverse	GAGAAGCACTGCATCCTCGT
GDF15	Forward	CAACCAGAGCTGGGAAGATTCG
GDF15	Reverse	CCCGAGAGATACGCAGGTGCA
HMGB2	Forward	TCTGAGGAAAAGCTCGCACC
HMGB2	Reverse	GCGTACGAGGACATTTTGCC
MARCKS	Forward	CGTTGGACCCCGCATCTTAT
MARCKS	Reverse	TCCCAGATTTGTAGCCGCAC
MMP1	Forward	TGTGGTGTCTCACAGCTTCC
MMP1	Reverse	CGCTTTTCAACTTGCCTCCC
NEAT1	Forward	GCTGGACCTTTCATGTAACGGG
NEAT1	Reverse	TGAACTCTGCCGGTACAGGGAA
PTMA	Forward	CAGGAGGCTGACAATGAGGT
PTMA	Reverse	GCTTGCCCGTAGCTGACTC
QSOX1	Forward	ACATGGCTGACCTGGAATCTGC
QSOX1	Reverse	GCAGGAAGTTCTGGACTAAGGG
RAB13	Forward	GACATCTTGCTCAAGTCAGGAGG
RAB13	Reverse	CAGGGAGCACTTGTTGGTGTTC
STC1	Forward	ACTCAGCTGAAGTGGTTCGT
STC1	Reverse	TTTCCAGGCATGCAAAAGCC
WFDC1	Forward	TTCTGTGTGCGTCTGGAAGG
WFDC1	Reverse	GGAGGAGAAGTAGCAAGAGGC

Supplementary Table 3. Percent composition of cells from each cluster calculated in each sample of senescence models illustrated in Figure 2A–2C.

Cluster	CTRL	RS	IR	ETO
**5**	17.04	7.32	0.38	0.18
**0**	62.85	24.43	0.86	0.77
**1**	16.13	36.39	15.58	19.67
**3**	3.47	12.35	28.74	15.29
**2**	0.30	9.13	23.52	30.87
**4**	0.20	8.20	22.76	25.47
**7**	0.00	0.74	3.22	5.16
**6**	0.02	1.45	4.94	2.59
