# Supplementary material for: Single-cell transcriptomic analysis uncovers diverse and dynamic senescent cell populations
Source: Aging (Albany NY). 2023 Apr 19;15(8):2824–51. doi: 10.18632/aging.204666 (PMC10188353; doi:10.18632/aging.204666)
Supplement: Supplementary Table 5 [file aging-15-204666-s005.pdf]

Supplementary Table 5. Percent composition of cells from each cluster calculated in each time-point of ETO time-course illustrated in Figure 3A.

Cluster	Day 0	Day 1	Day 2	Day 4	Day 7	Day 10
**6**	7.99	0.45	1.19	0.61	0.48	0.89
**1**	52.40	22.93	17.62	11.51	14.73	10.06
**4**	20.33	10.71	6.77	3.96	6.16	3.36
**0**	15.24	30.58	42.36	28.52	33.56	30.40
**2**	2.21	30.85	25.88	18.89	13.02	9.53
**3**	0.96	1.61	2.31	20.91	17.47	26.84
**5**	0.87	2.87	3.86	15.59	14.58	18.93
